# Effect of hydrophobic moment on membrane interaction and cell penetration of apolipoprotein E-derived arginine-rich amphipathic α-helical peptides

**DOI:** 10.1038/s41598-022-08876-9

**Published:** 2022-03-23

**Authors:** Yuki Takechi-Haraya, Takashi Ohgita, Mana Kotani, Hiroki Kono, Chihiro Saito, Hiroko Tamagaki-Asahina, Kazuchika Nishitsuji, Kenji Uchimura, Takeshi Sato, Ryuji Kawano, Kumiko Sakai-Kato, Ken-ichi Izutsu, Hiroyuki Saito

**Affiliations:** 1 Division of Drugs, National Institute of Health Sciences, 3-25-26 Tonomachi, Kawasaki-ku, Kawasaki, 210-9501 Japan; 2 Department of Biophysical Chemistry, Kyoto Pharmaceutical University, 5 Misasagi-Nakauchi-cho, Yamashina-ku, Kyoto, 607-8414 Japan; 3 Department of Biotechnology and Life Science, Tokyo University of Agriculture and Technology, 2-24-6 Naka-cho, Koganei, Tokyo 184-8588 Japan; 4 Division of Liberal Arts Sciences, Kyoto Pharmaceutical University, 1 Misasagi-Shichono-cho, Yamashina-ku, Kyoto, 607-8414 Japan; 5 Department of Biochemistry, Wakayama Medical University, 811-1 Kimiidera, Wakayama, 641-8509 Japan; 6 Unité de Glycobiologie Structurale et Fonctionnelle, UMR 8576 CNRS, Université de Lille, 59655 Villeneuve d’Ascq, France; 7 School of Pharmacy, Kitasato University, Shirokane 5-9-1, Minato-ku, Tokyo, 108-8641 Japan

**Keywords:** Membrane biophysics, Permeation and transport, Peptide delivery

## Abstract

We previously developed an amphipathic arginine-rich peptide, A2-17, which has high ability to directly penetrate across cell membranes. To understand the mechanism of the efficient cell-penetrating ability of the A2-17 peptide, we designed three structural isomers of A2-17 having different values of the hydrophobic moment and compared their membrane interaction and direct cell penetration. Confocal fluorescence microscopy revealed that cell penetration efficiency of peptides tends to increase with their hydrophobic moment, in which A2-17 L14R/R15L, an A2-17 isomer with the highest hydrophobic moment, predominantly remains on plasma cell membranes. Consistently, Trp fluorescence analysis indicated the deepest insertion of A2-17 L14R/R15L into lipid membranes among all A2-17 isomers. Electrophysiological analysis showed that the duration and charge flux of peptide-induced pores in lipid membranes were prominent for A2-17 L14R/R15L, indicating the formation of stable membrane pores. Indeed, the A2-17 L14R/R15L peptide exhibited the strongest membrane damage to CHO-K1 cells. Atomic force microscopy quantitatively defined the peptide-induced membrane perturbation as the decrease in the stiffness of lipid vesicles, which was correlated with the hydrophobic moment of all A2-17 isomers. These results indicate that optimal membrane perturbation by amphipathic A2-17 peptide is critical for its efficient penetration into cells without inducing stabilized membrane pores.

## Introduction

Arginine-rich peptides (ARPs) have attracted attention because of their ability to deliver various cargos into cells across the hydrophobic barrier imposed by cell membranes both in vitro and in vivo^1,2^. Two major types of mechanisms for the cellular entry of ARPs have been proposed in a case-by-case manner based on their physicochemical properties^1,3–6^. At physiological temperature, ARPs first bind to cell membranes, and afterward internalize into cells via proteins-involved dynamic membrane trafficking mechanisms including endocytosis or direct membrane penetration, or both. direct membrane penetration is essentially an energy- and receptor-independent physicochemical phenomenon accompanied by transient deformation of plasma lipid membranes. For a better methodology of cytosolic delivery of innovative therapeutic molecules such as proteins and nucleotides with avoiding the lysosomal degradation of drugs and cytotoxicity^7,8^, additional insight into the mechanism of the cell membrane penetration of ARPs is necessary.

One of the most promising strategies for developing the peptide sequence of highly membrane-penetrable ARPs is to exploit helical structures with an amphipathic interface that enhances their binding to lipid membranes and cell penetration. The amphipathicity of ARPs generally plays a crucial role in their membrane penetration^9,10^. We have previously developed an amphipathic α-helical peptide, A2-17, with a relatively high ability to directly penetrate cell membranes even at the low peptide concentration at which typical ARPs such as Tat, R8, and Rev do not exhibit efficient cell penetration^11,12^. Although A2-17 did not induce significant cytotoxicity in our experimental conditions, a previous study has reported that amphipathic ARPs exhibits similar properties to the membrane-active antimicrobial peptides^13^, which can cause stable membrane pores or destroy the lipid membrane barrier. In this regard, the relationship of the amphipathicity of peptides with their membrane interaction and cell membrane penetration ability is not well understood^12,14–17^.

In this study, to understand the cell penetration mechanism of A2-17 peptide in terms of peptide amphipathicity, we designed three structural isomers of A2-17 with different hydrophobic moment values—measures of α-helical peptide amphipathicity^18^. Comparison of the membrane interaction and direct cell penetration of the A2-17 isomers showed that the cell penetration efficiency of peptides tends to increase with their amphipathicity, but A2-17 L14R/R15L, an A2-17 isomer with the highest hydrophobic moment, predominantly remains on plasma cell membranes. Because the level of peptide-induced membrane perturbation required for direct cell penetration was correlated with the hydrophobic moment of all A2-17 isomers including the A2-17 L14R/R15L peptide, our results indicate that optimal plasma membrane perturbation by A2-17 is critical for its efficient direct penetration into cells.

## Results

### α-Helix-forming ability of A2-17 structural isomers

**Figure 1:**
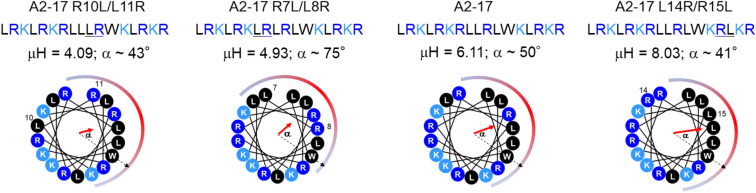
Structural isomers of amphipathic α-helical A2-17. The helical wheel diagrams for amino acid sequences of A2-17 R10L/L11R, A2-17 R7L/L8R, A2-17, and A2-17 L14R/R15L arranged as an ideal α-helix (100° rotation per residue) seen down the long axis from the amino-terminal end. The hydrophobic moment (μH), as a measure of amphipathicity of α-helix, for each peptide was calculated using the MPEx software (https://blanco.biomol.uci.edu/mpex/). The bold arrows represent hydrophobic moments as vectors. The hydrophobic domain of the helical wheel is shown by a semi-circle, at which a dotted arrow represents the position vector of Trp residue. The value α is the angle between the hydrophobic moment and the position vector of the Trp residue in the helical wheel diagram.

Based on the α-helical wheel diagram^19^, we designed A2-17 structural isomers with different hydrophobic moments (Fig. 1), in which the order of hydrophobic moment is as follows: A2-17 R10L/L11R < A2-17 R7L/L8R < A2-17 < A2-17 L14R/R15L.

**Figure 2:**
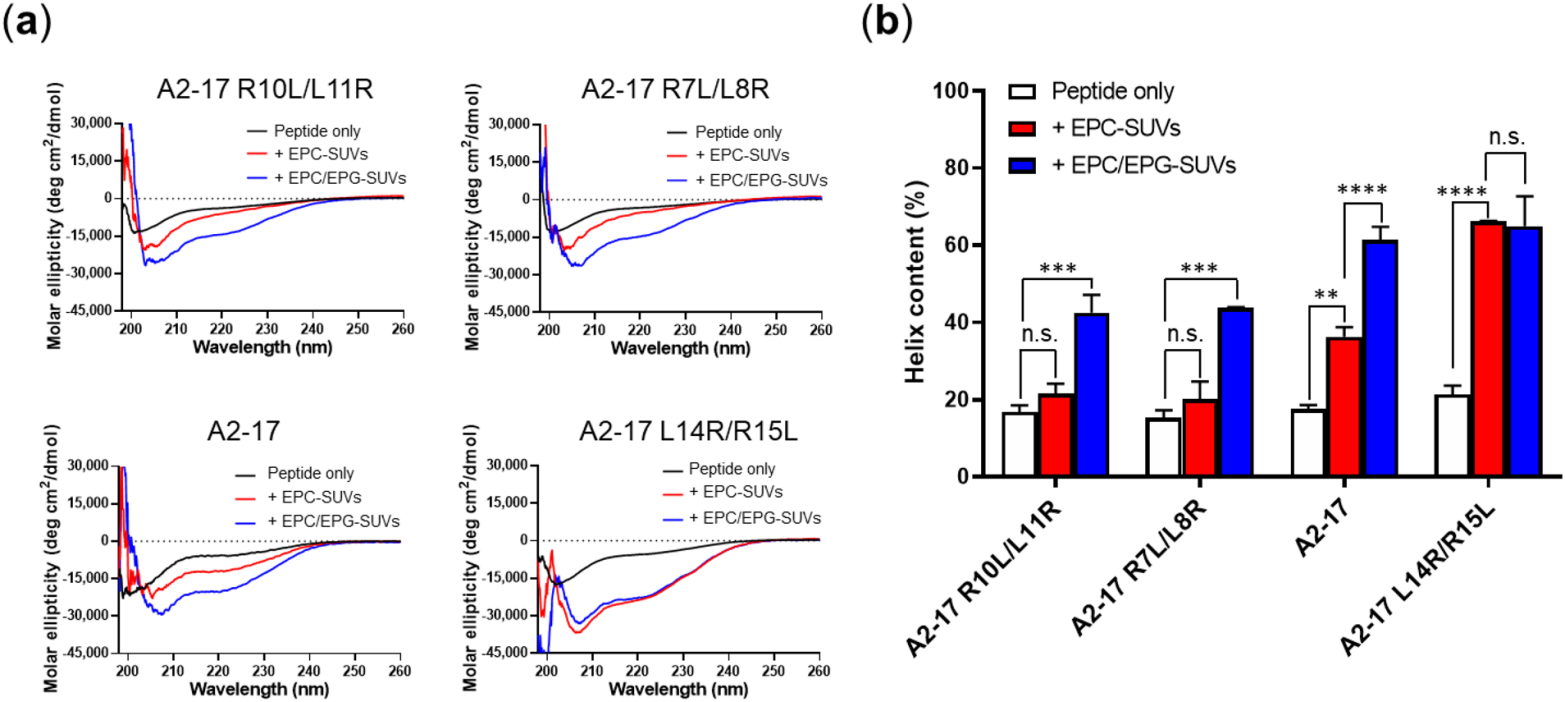
(**a**) Far-UV CD spectra of A2-17 structural isomers in the absence (Peptide only) or presence of lipid vesicles (+ EPC-SUVs or + EPC/EPG-SUVs; lipid/peptide molar ratio =  ~ 61) in 10 mM Tris buffer (150 mM NaCl, pH 7.4). Weight amounts of peptides that were determined by the BCA method may have slightly different peptide content, giving the variation in CD signal (black curve). (**b**) α-Helix contents of peptides in the absence (Peptide only) or presence of lipid vesicles (+ EPC-SUVs or + EPC/EPG-SUVs). Data for A2-17 are from our previous study^11^. **p < 0.01; ***p < 0.001; ****p < 0.0001; n.s., not significant.

Figure 2a shows circular dichroism (CD) spectra of peptides in the presence of small unilamellar vesicles (SUVs) as the cell membrane model. In our experiment, the lipid/peptide molar ratio was ~ 61. Because spectral distortion at wavelengths less than ~ 200 nm was caused by light scattering from the vesicles, we evaluated CD spectra at wavelengths over 200 nm according to the guideline^20^. A significant negative Cotton effect observed at 208 and 222 nm confirmed the actual α-helical conformation of all peptides bound to lipid membranes^20^. The ratio of the molar ellipticities at 222 nm and 208 nm is a detective indicator for intermolecular peptide–peptide interactions^21^. The ellipticity ratio was less than 1 for all A2-17 isomers, indicating that the peptides do not have the property of oligomerization via helix–helix interactions in the membrane. In the presence of egg phosphatidylcholine (EPC)-SUVs, the reinforcement of the α-helical structure with the membrane was not prominent for A2-17 R10L/L11R and A2-17 R7L/L8R, whereas A2-17 and A2-17 L14R/R15L exhibited more than two-fold increases in their α-helix content (Fig. 2b). This indicates that A2-17 and A2-17 L14R/R15L have an amphipathic nature that allows it to bind to neutrally charged lipid membranes through non-electrostatic interactions between the membrane and the hydrophobic residues of the non-polar face of the α-helix (Fig. 1) required for the effective binding of ARPs to cellular lipid membranes^11,22,23^. In contrast, the α-helical conformation of the peptides, except for A2-17 L14R/R15L, was more enhanced upon the interaction with EPC/egg phosphatidylglycerol (EPG)-SUVs than with EPC-SUVs (Fig. 2b), indicating that the electrostatic interaction between basic residues of the peptide and negatively charged lipids of EPC/EPG-SUVs further stabilizes the α-helical conformation of peptides. Such electrostatic interaction appears to have little effect on the lipid binding of the A2-17 L14R/R15L peptide.

### Cell membrane penetration of peptides

**Figure 3:**
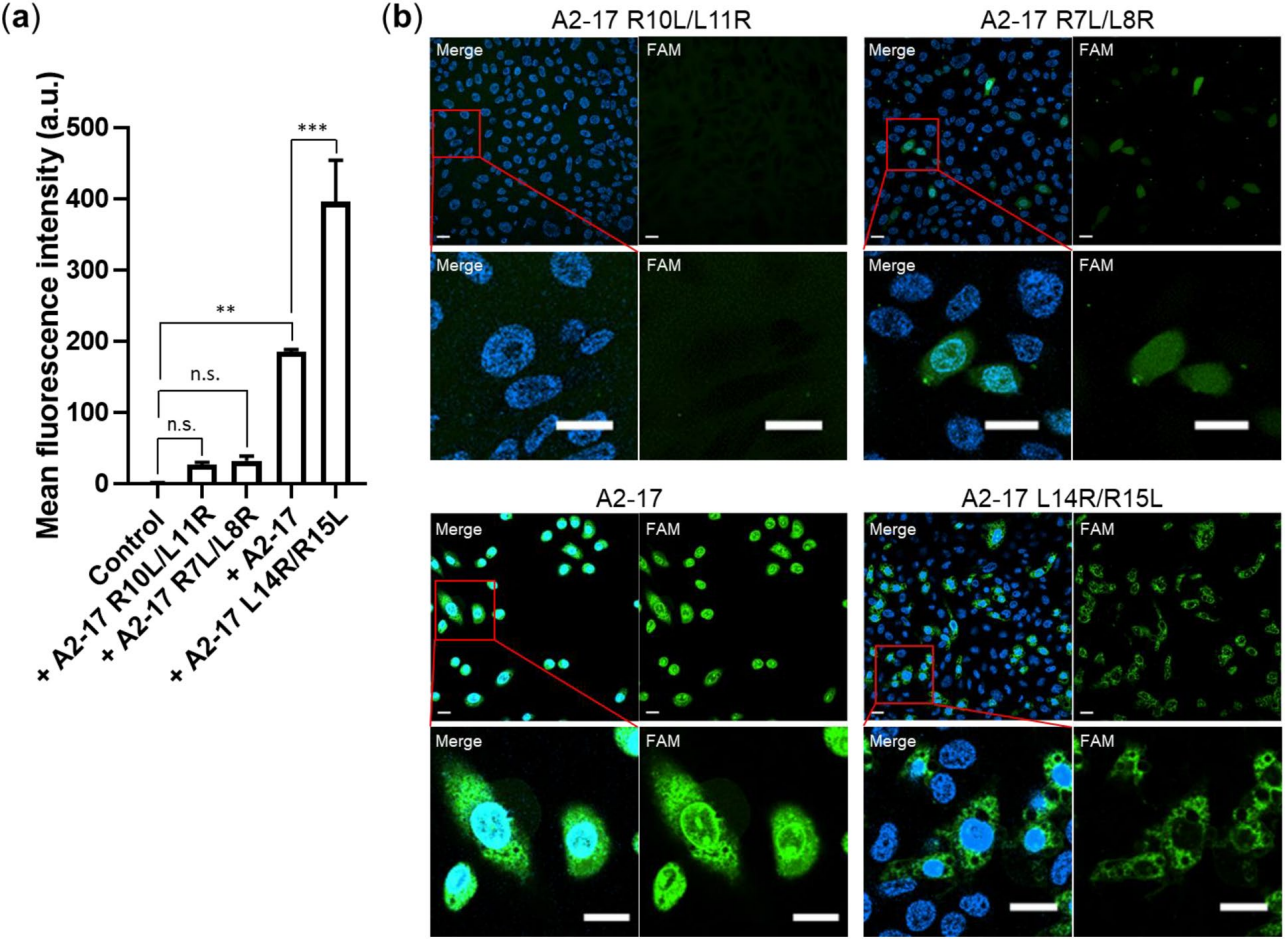
Analysis of cell membrane penetration of A2-17 structural isomers. (**a**) Flow cytometric quantification of the amount of cell-associated (membrane-bound and internalized) peptide in CHO-K1 cells treated with 2 µM of FAM-labeled peptides for 30 min at 4 °C. **p < 0.01; ***p < 0.001; n.s., not significant. (**b**) Confocal fluorescent images of CHO-K1 cells treated with 2 µM of FAM-labeled peptides for 30 min at 4 °C. FAM fluorescence (green) and Hoechst fluorescence (blue) counterstaining nuclei are shown in the merge image (Merge) along with the image of FAM fluorescence (FAM). The scale bars represent 20 μm.

To examine the direct cell penetration ability of A2-17 and its structural isomers, we next performed flow cytometric analysis and confocal laser scanning microscopic (CLSM) observation in Chinese hamster ovary (CHO)-K1 cells at 4 °C, where all the membrane trafficking pathways including endocytosis are strongly suppressed^24^. Flow cytometric analysis showed that the mean fluorescence intensity of the cells treated with 5(6)-carboxyfluorescein (FAM)-labeled peptides increased in the order of A2-17 R10L/L11R ≈ A2-17 R7L/L8R ≪ A2-17 < A2-17 L14R/R15L (Fig. 3a), indicating that increase in the hydrophobic moment of A2-17 isomers enhances their cell binding and penetration. Since flow cytometry measurements are sensitive to the amount of fluorescence-labeled peptides bound to cell membranes, it evaluates the quantity of cell-associated (membrane-bound and cell-internalized) peptide^25^. In contrast, the CLSM observation visualizes the cell-penetrating behavior of peptides (Fig. 3b). The fluorescence signal of peptides within the cells increased with peptide amphipathicity, especially for the A2-17 peptide, while the signal of A2-17 L14R/R15L peptide was localized at the peripheral region of the cell. This observation indicates that A2-17 L14R/R15L prefers to remain on plasma membranes despite high flow cytometric fluorescence intensity associated with the cells (Fig. 3a). A similar observation was noted under conditions where CHO-K1 cells were incubated with a higher concentration of FAM-labeled peptides (Fig. S1). These results indicate the preference of the A2-17 L14R/R15L peptide for hydrophobic membrane environment compared to A2-17.

### Trp fluorescence analysis for binding of peptides to lipid membrane

**Figure 4:**
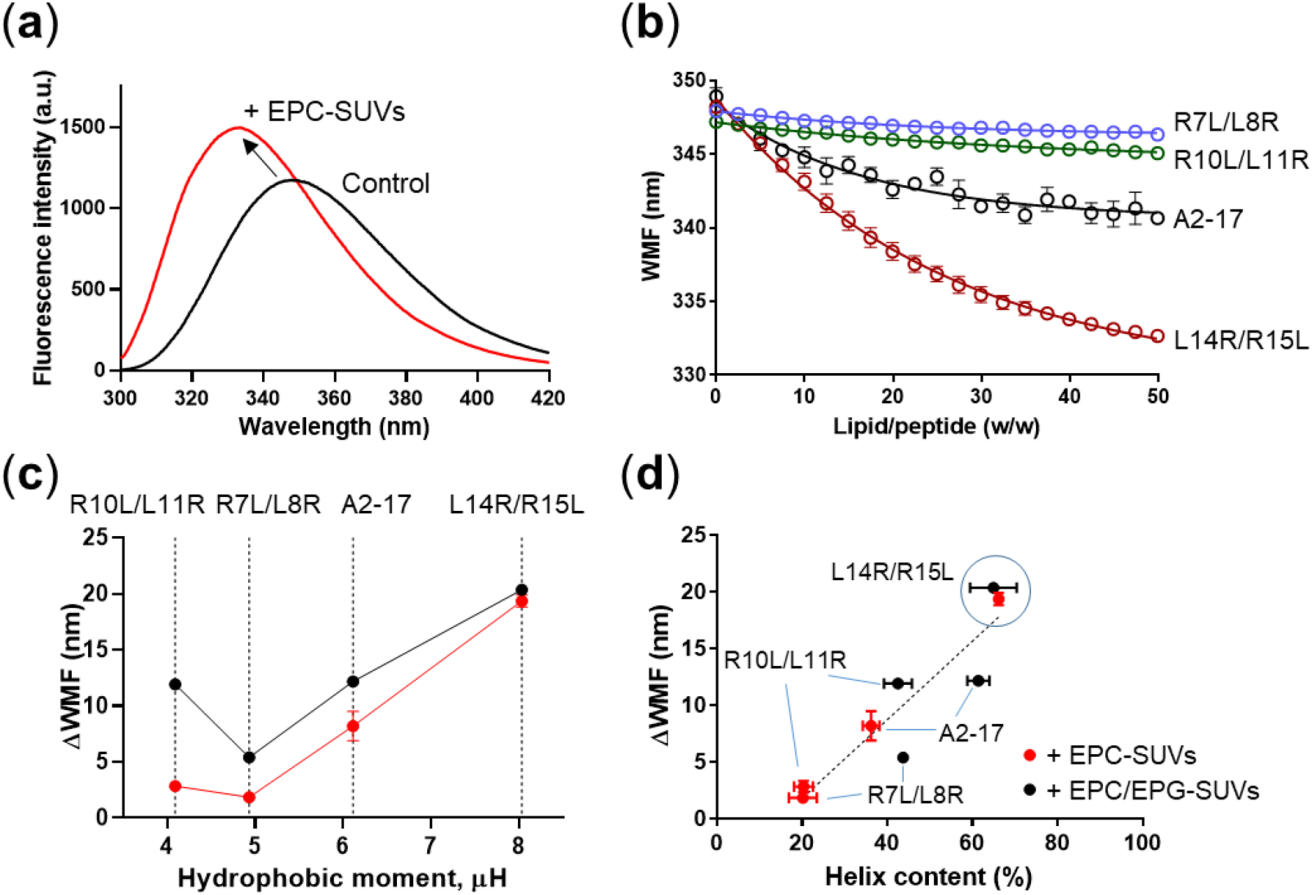
Trp fluorescence analysis of binding of A2-17 structural isomers to lipid membranes. (**a**) Trp fluorescence spectra of A2-17 L14R/R15L in the absence (Control) and presence of EPC-SUVs (+ EPC-SUVs). The concentrations of peptide and total lipid were 50 μg/mL and 1 mg/mL, respectively (lipid/peptide molar ratio =  ~ 61). (**b**) Changes in WMF of Trp residue for peptides as a function of weight ratio of total lipid of EPC-SUVs to peptide (lipid/peptide molar ratio changes from 0 to ~ 154). One-phase decay curve fitting using the least squares method was applied to obtain the blue shift of WMF at plateau (ΔWMF). The solid lines represent the best fit. (**c**) Plot of the ΔWMF against hydrophobic moment (μH) of peptides in the presence of EPC-SUVs (red dots) or EPC/EPG-SUVs (black dots). (**d**) Correlation of ΔWMF with α-helix content of peptides bound to EPC-SUVs (red dots) or EPC/EPG-SUVs (black dots). The dotted line represents the linear regression of the data (coefficient of determination *r*^2^ = 0.84). Data are expressed as mean ± S.E.

To investigate the lipid membrane interaction of peptides, which is essential for their cell membrane penetration, we performed Trp fluorescence measurement. Figure 4a shows typical Trp fluorescence spectra of A2-17 L14R/R15L in the absence and presence of EPC-SUVs. The Trp fluorescence typically exhibits an increased fluorescence intensity with a blue shift in the presence of lipid vesicles^26^. As shown in Fig. 4b, the wavelength of maximum fluorescence (WMF) that reflects the change in Trp environment decreased with increased lipid/peptide weight ratio, wherein A2-17 L14R/R15L exhibited the largest decrease. Based on the one-site binding model^11^, we confirmed that the value of the dissociation constant *K*_d_ for the binding of A2-17 L14R/R15L to EPC-SUVs was 1.4 ± 0.18 µM, which is comparable to that for the binding of A2-17 to EPC-SUVs (*K*_d_ = 1.1 ± 0.42 µM)^11^. The *K*_d_ values of A2-17 R10L/L11R and A2-17 R7L/L8R were difficult to determine accurately because of small changes in their fluorescence spectra.

The insertion degree of the peptide into lipid membranes was evaluated at conditions of peptides with an excess amount of lipid vesicles, where the change in WMF (ΔWMF) reaches a plateau. The WMF at the plateau state was determined by performing one-phase decay curve fitting of the data in Fig. 4b. Figure 4c compares ΔWMF values for all A2-17 isomers bound to EPC- or EPC/EPG-SUVs. A2-17 R10L/L11R and A2-17 R7L/L8R peptides exhibited a slight change in ΔWMF values upon binding to EPC-SUVs despite notable ΔWMF values in the case of EPC/EPG-SUVs, implying their relatively low binding affinities for lipid membranes. Since the concentration gradient of peptides across the membrane is the driving force for efficient lipid membrane penetration^27^, such a low lipid-binding ability may explain the disappearance or low efficiency of cell penetration of these peptides (Figs. 3, S1). In contrast, A2-17 and A2-17 L14R/R15L exhibited substantial ΔWMF values in either EPC-SUVs or EPC/EPG-SUVs (Fig. 4c), consistent with their efficient cell binding and penetration. For all A2-17 isomers, a simple correlation between ΔWMF and the hydrophobic moment of peptides was not apparent.

We also found a linear relationship of ΔWMF with the α-helix content of peptides bound to EPC- or EPC/EPG-SUVs (Fig. 4d), suggesting that the clustering of hydrophobic residues on the non-polar face of α-helix of A2-17 isomers promotes their membrane binding and insertion. In particular, A2-17 L14R/R15L exhibits the largest ΔWMF value and helix content bound to both EPC- and EPC/EPG-SUVs, indicating that the high α-helical hydrophobicity of A2-17 L14R/R15L greatly facilitates the insertion of the peptide into lipid membranes.

### Electrophysiological analysis for membrane penetration and pore formation of peptides

To investigate mechanisms underlying the lipid membrane penetration of A2-17 structural isomers without fluorescent labeling, electrophysiological measurement using a planar dioleoylphosphatidylcholine (DOPC) bilayer membrane was performed. Each current event can be classified into a “spike” or “long-lasting” signal based on the event duration, as described previously^28^. Peptides that exhibit the spike current signal (duration < 20 ms) might be advantageous for causing transient pore formation/destabilization of the lipid membrane, which is thought to be required for the cell membrane penetration of ARPs^5,6,28–30^. Indeed, the spike current signal is predominantly observed for random coiled or polyproline II helix cell-penetrating ARPs as demonstrated in our previous study^31^. The long-lasting current signal (duration ≥ 20 ms) is attributed to the formation of stable pores, frequently observed with antimicrobial pore-forming peptides^28^.

**Figure 5:**
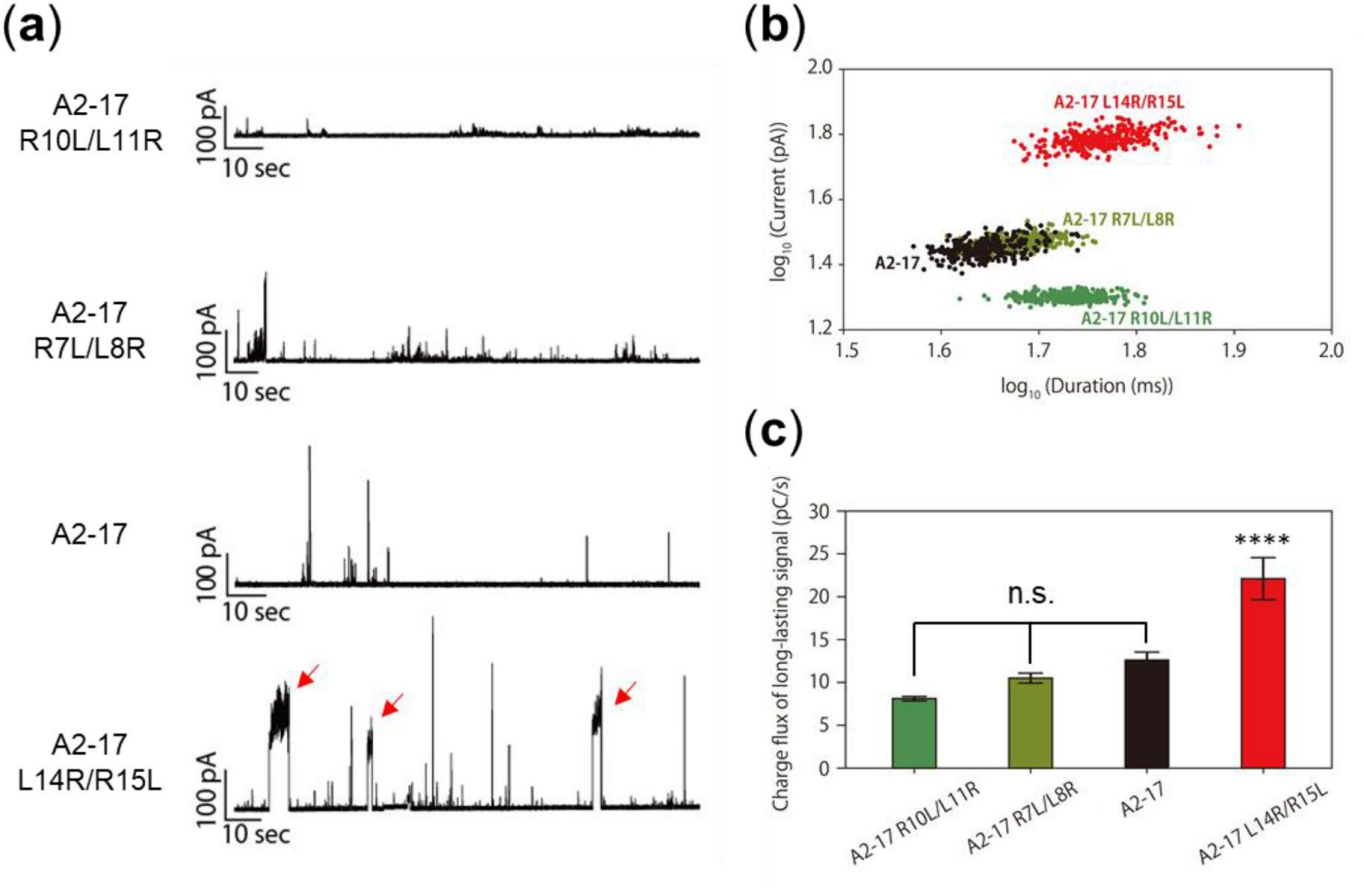
Analysis of the channel current signals of A2-17 structural isomers upon interaction with planar DOPC membranes. (**a**) Typical current and time traces of A2-17 R10L/L11R, A2-17 R7L/L8R, A2-17, and A2-17 L14R/R15L. A pattern of spike current signals with high current values was observed for A2-17, whereas A2-17 L14R/R15L showed long-lasting current signals even with second-order duration as indicated by arrow. The concentration of peptide was 100 nM and the calculated molar ratio of lipid on the droplet surface to peptide was ~ 67 (see the Methods for more detail). (**b**) Plot of current versus duration of long-lasting (stable pore) signals caused by A2-17 R10L/L11R (dark green), A2-17 R7L/L8R (light green), A2-17 (black), and A2-17 L14R/R15L (red). (**c**) The charge flux of long-lasting signals caused by peptides. ****p < 0.0001, compared with A2-17 R10L/L11R. n.s., not significant.

Figure 5a shows the typical current‒time traces for samples after the peptide addition; a pattern of spike current signals with higher current values was observed for A2-17 compared with A2-17 R10L/L11R and A2-17 R7L/L8R. A2-17 L14R/R15L exhibited long-lasting current signals even with second-order duration (as indicated by arrow) in addition to spike current signals. The current events classified as spike signals were predominant for A2-17 and A2-17 L14R/R15L. The spike signal ratio increased in the order of A2-17 R10L/L11R (41.3%) < A2-17 R7L/L8R (44.3%) < A2-17 L14R/R15L (62%) < A2-17 (73.3%). These results imply that among all the peptides studied, A2-17 has the most favorable characteristics for membrane penetration in the cell membrane model.

We also focused exclusively on peptide-induced long-lasting current events, and analyzed the distribution of the current and duration of each long-lasting signal. As shown in Fig. 5b, the duration time was longer in the order of A2-17 < A2-17 R7L/L8R < A2-17 R10L/L11R < A2-17 L14R/R15L. In addition, the current induced by A2-17 L14R/R15L was much larger than that of the other isomers at similar values of duration. Consistently, the total charge flux of the long-lasting pores induced by A2-17 L14R/R15L is much larger than that of the other isomers (Fig. 5c). These results indicate that A2-17 L14R/R15L causes larger-sized, stable membrane pores and mediates the permeation of a greater number of ions, ionic molecules, or both, compared with the other isomers.

### Atomic force microscopic (AFM) analysis for peptide-induced membrane perturbation

**Figure 6:**
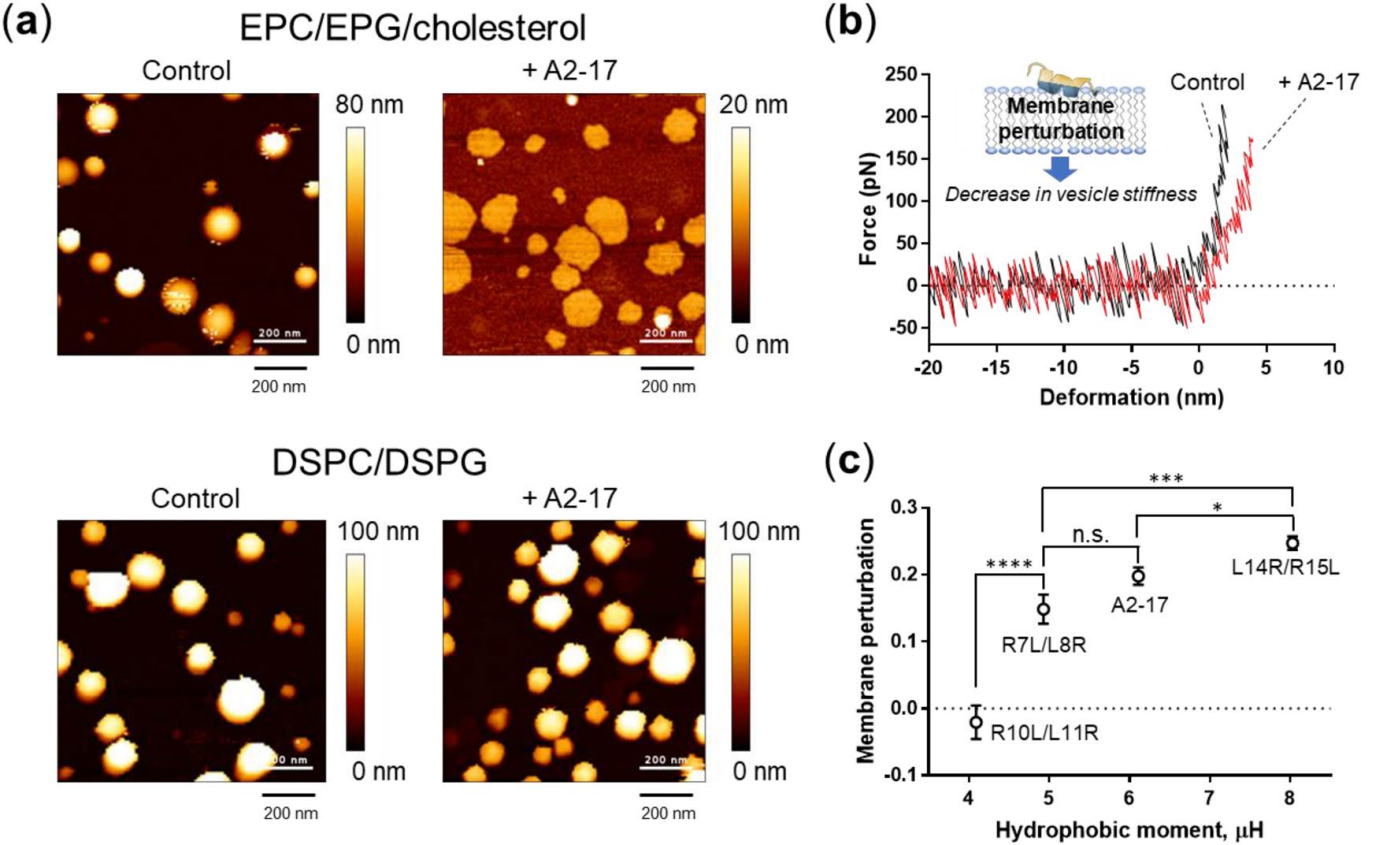
AFM analysis of lipid membrane perturbation caused by A2-17 structural isomers at a lipid/peptide molar ratio of 1. (**a**) AFM images of EPC/EPG/cholesterol-LUVs and DSPC/DSPG-LUVs in the absence (Control) and presence of A2-17 (+ A2-17). Scale bars represent 200 nm. (**b**) Representative force–deformation curves for DSPC/DSPG-LUVs in the absence (Control) and presence of A2-17 (+ A2-17). Membrane perturbation by peptides decreases the slope of the curve, that is, the lipid vesicle stiffness. (**c**) Correlation of membrane perturbations of DSPC/DSPG-LUVs caused by peptides with their hydrophobic moment (μH) values. *p < 0.05; ***p < 0.001; ****p < 0.0001; n.s., not significant.

We further performed AFM analysis to investigate peptide-induced membrane perturbation as a result of peptide binding to lipid membranes. Figure 6a presents AFM images of EPC/EPG/cholesterol-large unilamellar vesicles (LUVs) and distearoylphosphatidylcholine (DSPC)/distearoylphosphatidylglycerol (DSPG)-LUVs in the absence and presence of A2-17. An addition of peptide caused collapse of EPC/EPG/cholesterol-LUVs into membrane patches with a height of ~ 5 nm, which corresponds to the thickness of lipid bilayers^32^. A lipid vesicle on a solid substrate has been shown to expand its spherical morphology to a lipid bilayer patch when vesicle stiffness cannot endure the traction stress derived from the adhesion energy of the vesicle with the substrate^32,33^. Therefore, deformation of EPC/EPG/cholesterol-LUVs by the addition of A2-17 indicates a decrease in the stiffness of lipid vesicles. On the other hand, DSPC/DSPG-LUVs in the presence of peptides maintained their spherical morphology.

Using DSPC/DSPG-LUVs, we obtained force–deformation curves (Fig. 6b), in which the slope of the force–deformation curve represents the stiffness of a lipid vesicle: the more densely the lipids pack in the membrane, the larger the stiffness value^34^. Therefore, the decrease in slope by the addition of A2-17 indicates the peptide-induced destabilization of lipid packing in the membrane. We defined this peptide-induced membrane perturbation as the decrease in the lipid vesicle stiffness using (*S*_control_ − *S*)/*S*_control_, where *S*_control_ and *S* are the stiffnesses of control lipid vesicles and lipid vesicles with peptide, respectively. The plot of membrane perturbation values for the A2-17 isomers against the hydrophobic moment (Fig. 6c) distinctly demonstrated that the membrane perturbation level increases with the peptide hydrophobic moment.

## Discussion

We have previously demonstrated that the amphipathic α-helical peptide A2-17 exhibits a higher affinity for lipid membranes and deeper insertion into lipid membrane interiors, resulting in greater efficiency of cell membrane penetration compared with non-amphipathic ARPs such as Tat, polyarginines, and Rev^11,12^. The proposed mechanism underlying the direct membrane penetration of highly charged non-amphipathic ARPs is that they penetrate lipid membranes through the hydrophilic domain of transient membrane defects or pores driven by the interaction between arginine residues in peptides and phosphate groups of lipids^35–38^. It is possible that the generation of such transient hydrophilic domain is enhanced by membrane perturbation through the disturbance of lipid-lipid interactions caused by deeper insertion of amphipathic α-helical ARPs into the membrane interiors. To support this idea, electrophysiological analysis (Fig. 5) suggested that the A2-17 and A2-17 L14R/R15L peptides predominantly cause transient membrane destabilization (spike current signal ratio is 73.3% for A2-17 and 62% for A2-17 L14R/R15L). Consistently, our AFM results showed that the membrane perturbation caused by amphipathic α-helical A2-17 structural isomers is closely correlated with their hydrophobic moment (Fig. 6c). In addition, the finding that A2-17 exhibited a much higher cell penetration efficiency than A2-17 R10L/L11R and A2-17 R7L/L8R (Figs. 3, S1) indicates that the amphipathic α-helix nature of A2-17 isomers is likely to contribute to efficient cell penetration. The relatively high cell penetration ability of A2-17 among the isomers was also observed at 37 °C (Fig. S2), indicating the efficient cell penetration of A2-17 at physiological temperature^11^.

The increased hydrophobic moment of A2-17 L14R/R15L compared with that of A2-17 strengthened α-helix-forming and membrane insertion ability (Figs. 2, 4). However, the A2-17 L14R/R15L peptide preferred to remain on cell plasma membranes despite exhibiting the great cell penetration ability comparable to A2-17 (Figs. 3, S1). It is known that cell-penetrating ARPs can act as membrane-active antimicrobial peptides (AMPs), which induce stable pores or destroy the lipid membrane barrier^16,39,40^. Given that large amphipathicity and hydrophobicity of typical AMPs compared with those of ARPs are thought to stabilize and prolong the membrane pore^35,41,42^, A2-17 L14R/R15L may partially have the nature of AMPs. Indeed, the duration time and charge flux of stable membrane pores induced by A2-17 L14R/R15L were much larger than those by the other isomers (Fig. 5b, c). Therefore, our results indicate that the increase in amphipathicity of α-helical ARPs does not always improve the cell penetration ability, rather can enhance the cytotoxicity related to destabilized integrity of the cell membrane. Perhaps, a trade-off between membrane penetration and integrity should be considered: the physical perturbation required to generate the hydrophilic domain through which peptide molecules permeate into cells is likely to be moderate to prevent the formation of stable membrane pores or critical membrane damage. This may explain previous contradictory observations regarding cationic peptides, in which tuning helical hydrophobicity of amphipathic peptides did not always enhance their cell penetration, resulting in the amphipathicity-independent cellular uptake of peptides^8,14,15,43,44^.

The AFM method used in this study can evaluate peptide-induced membrane perturbation in direct and quantitative manners by measuring the stiffness of lipid vesicles. Our finding (Fig. 6c) indicates that the membrane perturbation caused by amphipathic α-helical A2-17 structural isomers is closely correlated with their hydrophobic moment, that is, their membrane binding and secondary structure properties. This suggests that A2-17 penetrates directly into cells accompanied by optimal plasma membrane perturbation, but not by stabilized pores in the membrane. To support this, the critical cytotoxicity in CHO-K1 cells as determined via the 3-[4,5-dimethylthiazol-2-yl]-2,5 diphenyl tetrazolium bromide (MTT) assay was not observed for all peptides, whereas the lactate dehydrogenase (LDH) assay detected cell membrane defects for the cells incubated with A2-17 L14R/R15L, and to a lesser extent with A2-17 (Fig. S3). Such peptide-induced LDH leakage has been reported for ARPs in a concentration-dependent manner^45^.

Since Trp fluorescence reflects the environment surrounding the Trp residue of a peptide, it is widely used for the evaluation of peptide insertion into lipid membranes, which is related to membrane perturbation^46–48^. However, a simple correlation between ΔWMF for Trp fluorescence and the hydrophobic moment of peptides was not observed (Fig. 4c). The Trp residue of A2-17 R10L/L11R is positioned in the hydrophobic domain that contains three arginine residues in the peptide α-helix, whereas the hydrophobic domain of A2-17 contains two arginine residues with the Trp residue (Fig. 1). Therefore, although the hydrophobic moment of A2-17 R10L/L11R is the lowest among the A2-17 isomers, the Trp residue of this peptide is possibly localized at the hydrophobic region of the membrane when electrostatic interactions with membranes play a crucial role. This may explain the similar values of ΔWMF for A2-17 R10L/L11R and A2-17 in the presence of EPC/EPG-SUVs. For A2-17 R7L/L8R, the Trp residue is positioned far from the hydrophobic domain in α-helix structure (Fig. 1); this structural feature would explain the lowest values of ΔWMF among the isomers either in the presence of EPC- or EPC/EPG-SUVs. Thus, the Trp fluorescence of amphipathic ARPs does not always give direct information about peptide insertion into lipid membranes.

In conclusion, by comparing the structural isomers of the amphipathic α-helical peptide A2-17 that have different hydrophobic moments, we have demonstrated that the A2-17 peptide has an optimal amphipathicity for membrane perturbation, leading to the efficient penetration of peptide across cell membranes. Further increase in the amphipathicity of A2-17 caused higher membrane perturbation related to cell membrane damage and predominant remaining on plasma cell membranes. We also found that the order of the peptide-induced membrane perturbation estimated from the stiffness of vesicles on AFM measurements is closely correlated with the hydrophobic moment of the peptides. These results indicate that the efficient cell penetration of A2-17 accompanies optimal plasma membrane perturbation, but not the formation of stabilized pores in the membrane.

## Methods

### Materials

EPC was purchased from Kewpie Corporation (Tokyo, Japan). EPG was purchased from NOF Corporation (Tokyo, Japan). DOPC and DSPC were purchased from Avanti Polar Lipids Ltd (Alabaster, AL, USA). DSPG was purchased from Cayman Chemical Company (Ann Arbor, MI, USA). Cholesterol was purchased from Sigma-Aldrich (St. Louis, MO, USA). A2-17 and its isomers were prepared via solid-phase synthesis method using Fmoc chemistry. The amino and carboxyl termini of each peptide were acetylated and amidated, respectively. The peptide sequences were as follows: A2-17 R10L/L11R, LRKLRKRLLLRWKLRKR; A2-17 R7L/L8R, LRKLRKLRLRLWKLRKR; A2-17, LRKLRKRLLRLWKLRKR; A2-17 L14R/R15L, LRKLRKRLLRLWKRLKR. The MPEx software v.3.3 (https://blanco.biomol.uci.edu/mpex/) was used to calculate the hydrophobic moment (µH) of the peptides, assuming that the helix structure was formed with an acetylated amino-terminal and amidated carboxyl-terminal, using the Wimley–White interfacial scale^49^. For the confocal fluorescent microscopic detection of peptides, the amino terminus of the peptide was labeled with FAM via a glycylglycine linker. Purification of synthesized peptides was carried out by reverse-phase liquid chromatography. The peptides were eluted with a linear gradient of acetonitrile and water containing 0.1% trifluoroacetic acid with detection at 220 nm. The purity of each eluted peptide was confirmed to be > 95% by reverse-phase liquid chromatography and MALDI-TOF mass spectrometry (Figs. S4–S11). The peptide concentrations were measured using Pierce™ BCA Protein Assay Kit according to the manufacturer’s instruction (Thermo Fisher Scientific, Waltham, MA, USA), where a known amount of each peptide was used as a standard. As the average molecular weight are identical among the A2-17 isomers, we used the weight unit rather than the molar unit of peptide for the convenience of experiments.

### Preparation of lipid vesicles

SUVs were prepared as previously described^50^. Briefly, a dried lipid film of EPC or EPC/EPG (4:1 molar ratio) was hydrated in 10 mM Tris buffer containing 150 mM NaCl (pH 7.4) and sonicated on ice under nitrogen. After removing titanium debris, the samples were centrifuged in a Beckman 70.1 Ti rotor at 40,000 rpm for 1.5 h at 15 °C to separate any remaining large vesicles.

LUVs were prepared as described^51^. Briefly, a dried lipid film of EPC/EPG/cholesterol (4:1:2.2 molar ratio) or DSPC/DSPG (4:1 molar ratio) was hydrated with 10 mM Tris buffer containing 150 mM NaCl (pH 7.4) under mechanical agitation for 5 min at 60 °C. The resultant suspension was freeze-thawed five times using dry ice-methanol slush and a water bath of 60 °C, followed by extrusion 21 times through a mini-extruder equipped with a 0.1-μm polycarbonate filter (Avanti Polar Lipids, Alabaster, AL, USA). The extrusion was performed at 60 °C for EPC/EPG/cholesterol-vesicles or 70 °C for DSPC/DSPG-vesicles.

### CD measurements

Far-UV CD spectra were recorded from 190 to 260 nm at 25 °C using a J-1500 spectropolarimeter (JASCO, Tokyo, Japan) with a quartz cuvette of 1-mm path length. Peptide solutions (50 µg/mL) in 10 mM Tris buffer containing 150 mM NaCl (pH 7.4) in the absence and presence of lipid vesicles (1 mg/mL) were subjected to CD measurements. At this condition, the lipid/peptide molar ratio of the peptide-vesicle mixture was ~ 61. Each CD spectrum of the peptide sample was corrected by subtracting the corresponding baseline for the same concentration of lipid vesicles in the Tris buffer solution. The α-helix content of peptide was determined from the mean residue ellipticity at 222 nm, as described by Scholtz et al.^52^.

### Trp fluorescence measurements

Fluorescence measurements were carried out using an F-7000 fluorescence spectrophotometer (Hitachi, Tokyo, Japan) at 25 °C in 10 mM Tris buffer containing 150 mM NaCl (pH 7.4), as described previously^11^. Trp emission fluorescence spectra of 50 µg/mL of A2-17 R10L/L11R, A2-17 R7L/L8R, A2-17, or A2-17 L14R/R15L were recorded from 300 to 420 nm using a 290-nm excitation wavelength in the absence and presence of lipid vesicles. Each Trp fluorescence spectrum of the peptide was corrected by subtracting the baseline for the same concentration of lipid vesicles in buffer solution.

### CLSM

CLSM observation via the z-stack imaging mode was performed on an LSM 800 (Carl Zeiss, Oberkochen, Germany) with a C-Apochromat 40×/1.20 W Korr objective at an excitation wavelength of 488 nm for the visualization of FAM-labeled peptides as described^11^. CHO-K1 cells (2 × 10^5^ cells) were plated in a 35-mm glass-bottom dish coated with poly-L-lysine (Matsunami Glass Ind. Ltd., Osaka, Japan) and were incubated in nutrient F-12 Ham (Sigma, St. Louis, MO, USA) supplemented with 10% fetal bovine serum (FBS; Lonza Group, Basel, Switzerland). After incubation for 24 h (37 °C, 5% CO_2_), the cells were incubated with FAM-labeled peptides for 30 min at 4 or 37 °C in FBS-free F12-Ham medium. After the incubation, the cells were washed thrice with phosphate-buffered saline (PBS) on ice and stored in PBS, followed by confocal microscopic imaging. The nuclei of cells were counterstained with Hoechst 33342 (Life Technologies, Waltham, MA, USA), following the manufacturer's instructions, and visualized at an excitation wavelength of 405 nm. Throughout image acquisition, laser intensity, photomultiplier detector sensitivity and pinhole aperture values were kept constant.

### Flow cytometric analysis

The amount of cell-associated and internalized peptides in CHO-K1 cells was quantified via the flow cytometric method using a FACS-Calibur flow cytometer equipped with the Cell Quest Pro software v.4.02 (BD Biosciences), as described previously^11,31^. Briefly, cultured cells (1 × 10^5^ cells/well) in a 24-well plate were incubated in FBS-free F12-Ham medium with or without FAM-labeled peptides for 30 min at 4 or 37 °C. After incubation, the cells were washed with PBS and treated with 0.25% trypsin–EDTA for 5 min at 37 °C. Then, the cells were washed with PBS containing 2% FBS and collected by centrifugation. The resultant cell pellet was resuspended in PBS containing 2% FBS, and passed through a 25 μm-meshed filter, followed by flow cytometry analysis. FAM fluorescence at an excitation wavelength of 488 nm was monitored with 530/30 bandpass filter, and mean fluorescence values were determined from histograms.

### Cytotoxicity assay

Cytotoxicity of peptide in CHO-K1 cells was evaluated using the same MTT assay previously reported^31^. The LDH assay was also performed using a Cyto Tox-One homogenous membrane integrity assay kit according to the manufacturer’s recommendations (Promega Corp., Madison, WI, USA). Results are presented as percentages of the values of the control sample without the addition of peptides.

### Channel current analysis for membrane penetration of peptides

Electrophysiological measurement using a microfabricated device was performed as previously reported^53^. Briefly, planar lipid bilayers were prepared by the “droplet contact method” using a device, which was fabricated to have a set of two chambers with microfabrication technology. In this method, two lipid monolayers contact and form a planar lipid bilayer. First, DOPC (lipid/n-decan, 10 mg/mL) solution (0.7 µL) was poured into both chambers. Next, a droplet of 4.7 µL buffer solution (150 mM KCl, 10 mM MOPS, pH 7.0) was poured into both chambers. The peptide was dissolved in only one side of the chambers at 100 nM, in which the final concentration of lipid in the droplet was 1.89 mM (the actual molar lipid on the droplet surface measured against total molar peptide added was calculated to be ~ 67; voltage applying side). After adding the buffer solution, two lipid monolayers were contacted and formed a bilayer within a few minutes. The channel current value was then monitored using a JET patch-clamp amplifier (Tecella, Foothill Ranch, CA) connected to an Ag/AgCl electrode in each chamber. The channel current signals were detected using a 4-kHz low-pass filter at a sampling frequency of 20 kHz. Analysis of channel signals was performed using pCLAMP ver. 10.7 (Molecular Devices, CA, USA). Data obtained in this measurement are *n* (number of current signals) 600 > *n* > 150; *N* (number of experiments) > 3.

### AFM

AFM at 25 ± 1 °C in 10 mM Tris buffer containing 150 mM NaCl (pH 7.4) using a BioLever mini cantilever (BL-AC40TS, Olympus Co., Tokyo, Japan) via the QI mode of a JPK Nanowizard Ultra Speed microscope equipped with the Data Processing JPK software v.6.0 (JPK Instruments AG, Berlin, Germany) was performed by a slight modification of our previous procedure^54^. This method can directly measure the stiffness of a lipid vesicle immobilized on a solid substrate in an aqueous environment while simultaneously imaging the vesicle. Briefly, 200 μL of EPC/EPG/cholesterol-LUVs or DSPC/DSPG-LUVs (50 μM of total lipid) in Tris buffer solution was incubated on an aminopropyl-modified mica substrate for 20 min, and an additional 1.4 mL of Tris buffer solution was added, followed by AFM measurement. We chose these lipid compositions for lipid vesicle immobilization on the substrate, which is the main limitation of this method as discussed previously^34,51,54^. For the AFM measurement of peptide samples, an additional 1.4 mL of the Tris buffer solution containing peptide was added so that the final peptide/lipid molar ratio was 1. The cantilevers were routinely cleaned by ultraviolet-ozone treatment for 20 min using an ASM401 OZ (Asumi Giken, Ltd., Tokyo, Japan), rinsed with methanol and water, and dried in air. Prior to the measurements, a cantilever was calibrated in the air via the thermal noise method^55,56^, and immersed in the liquid sample for 10 min to thermally equilibrate. AFM images at a resolution < 8 nm/pixel were recorded with a 0.2–0.25 nN set point, a z-length of 100 nm, and a 15 μm/s extend/retract speed. Acquired AFM images of the lipid vesicles were analyzed using the Gwyddion software v.2.47 to measure the maximum height of each vesicle^57^. To obtain lipid vesicle stiffness, a linear fit was performed over the linear region of the force–deformation curve at the center of a lipid vesicle using the JPK Software. Prior to the stiffness data acquisition, tip cleanliness was checked by monitoring the force curve on the substrate, as described previously^51,58^. Because relatively small lipid vesicles tend to exhibit relatively great stiffness attributed to a relatively high membrane curvature^59^, we confirmed that comparison groups analyzed by AFM show no significant differences in height distributions (85 ± 24 nm). We defined the peptide-induced membrane perturbation as the decrease in the lipid vesicle stiffness using (*S*_control_ − *S*)/*S*_control_, where *S*_control_ and *S* are the stiffnesses of control lipid vesicles and lipid vesicles treated with peptide, respectively. Using the average stiffness for *S*_control_, we evaluated the membrane perturbation of lipid vesicles with peptide. Number of stiffness value *n* obtained in this measurement is 184 > *n* > 86 and the experiment was repeated thrice.

### Statistical analysis

The results are presented as the mean ± SE. Statistical analysis was performed using the GraphPad prism software v.9.3.1 (GraphPad Software, La Jolla, CA). The differences between groups were analyzed by one-way ANOVA with Tukey's multiple comparisons test. Results were considered statistically significant at a *p* value < 0.05.

## Supplementary Information

Supplementary Figures.
