# Supplementary material for: Effect of hydrophobic moment on membrane interaction and cell penetration of apolipoprotein E-derived arginine-rich amphipathic α-helical peptides
Source: Sci Rep. 2022 Mar 23;12:4959. doi: 10.1038/s41598-022-08876-9 (PMC8943082; doi:10.1038/s41598-022-08876-9)
Supplement: Supplementary file 1 — Supplementary Figures. [file 41598_2022_8876_MOESM1_ESM.pdf]

Supplementary Information (Figures S1-S11)

Effect of hydrophobic moment on membrane interaction and cell penetration of apolipoprotein E-derived arginine-rich amphipathic α-helical peptides

Yuki Takechi-Haraya[1,*], *Takashi Ohgita*[2], *Mana Kotani*[2], *Hiroki Kono*[2], *Chihiro Saito*[3], *Hiroko Tamagaki-Asahina*[4], *Kazuchika Nishitsuji*[5], *Kenji Uchimura*[6], *Takeshi Sato*[4], *Ryuji Kawano*[3], *Kumiko Sakai-Kato*[7], *Ken-ichi Izutsu*[1], *and Hiroyuki Saito*[2]

[1]Division of Drugs, National Institute of Health Sciences, 3-25-26 Tonomachi, Kawasaki-ku, Kawasaki 210-9501, Japan, [2]Department of Biophysical Chemistry, Kyoto Pharmaceutical University, 5 Misasagi-Nakauchi-cho, Yamashina-ku, Kyoto 607-8414, Japan, [3]Department of Biotechnology and Life Science, Tokyo University of Agriculture and Technology, 2-24-6 Naka-cho, Koganei, Tokyo 184-8588, Japan, [4]Division of Liberal Arts Sciences, Kyoto Pharmaceutical University, 1 Misasagi-Shichono-cho, Yamashina-ku, Kyoto 607-8414, Japan, [5]Department of Biochemistry, Wakayama Medical University, 811-1 Kimiidera, Wakayama 641-8509, Japan, [6]Unité de Glycobiologie Structurale et Fonctionnelle, UMR 8576 CNRS, Université de Lille, 59655 Villeneuve d'Ascq, France, [7]Kitasato University, Shirokane 5-9-1, Minato-ku, Tokyo 108-8641, Japan

[*]Corresponding author: haraya@nihs.go.jp

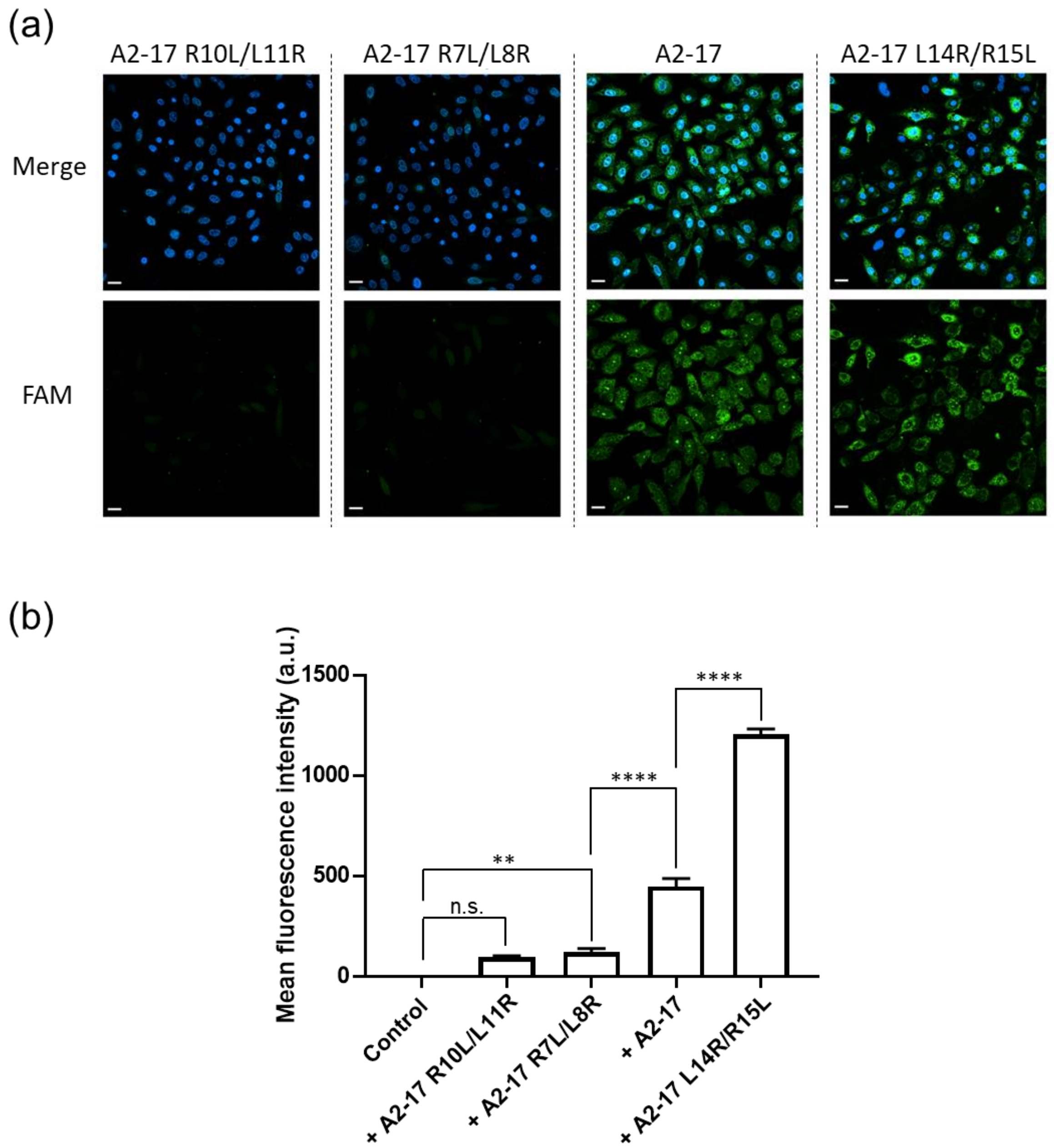

Figure S1. Analysis of cell membrane penetration of A2-17 structural isomers at 5 μM peptide concentration. (a) Confocal fluorescent images of CHO-K1 cells treated with FAM-labeled peptides for 30 min at 4 °C. FAM fluorescence (green) and Hoechst fluorescence (blue) counterstaining nuclei are shown in the merge image (upper panel) along with the image of FAM fluorescence (lower panel). The scale bars represent 20 μm. (b) Flow cytometric quantification of the amount of cell-associated (membrane-bound and internalized) peptide in CHO-K1 cells treated with FAM-labeled peptides for 30 min at 4 °C. **$p < 0.01$; ****$p < 0.0001$; n.s., not significant.

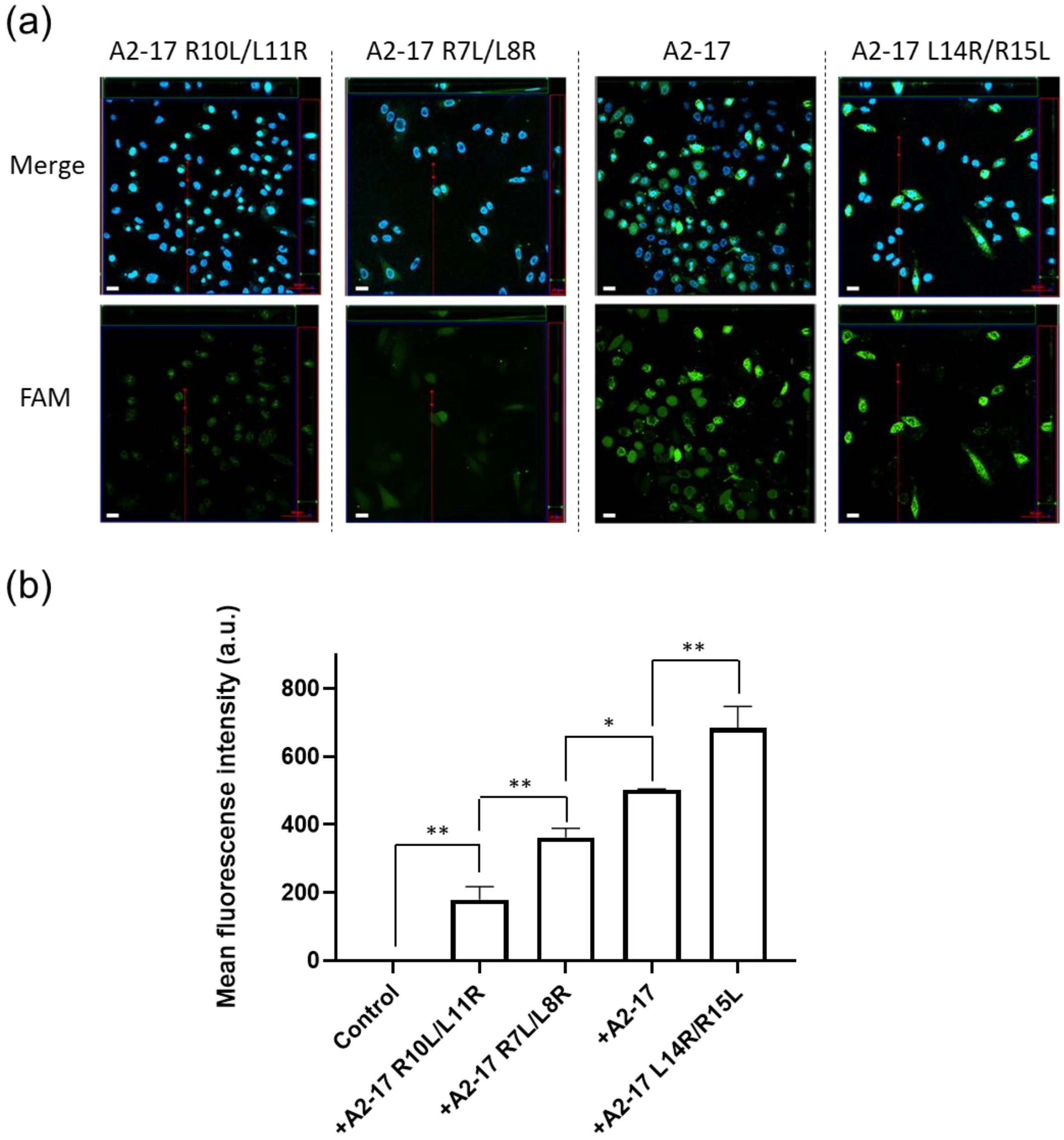

Figure S2. Analysis of cellular internalization of A2-17 structural isomers at physiological temperature. (a) Confocal fluorescent images of CHO-K1 cells treated with 2 μM FAM-labeled peptides for 30 min at 37 °C. FAM fluorescence (green) and Hoechst fluorescence (blue) counterstaining nuclei are shown in the merge image (upper panel) along with the image of FAM fluorescence (lower panel). The scale bars represent 20 μm. (b) Flow cytometric quantification of the amount of cell-associated (membrane-bound and internalized) peptide in CHO-K1 cells treated with 2 μM FAM-labeled peptides for 30 min at 37 °C. *$p < 0.05$; **$p < 0.01$.

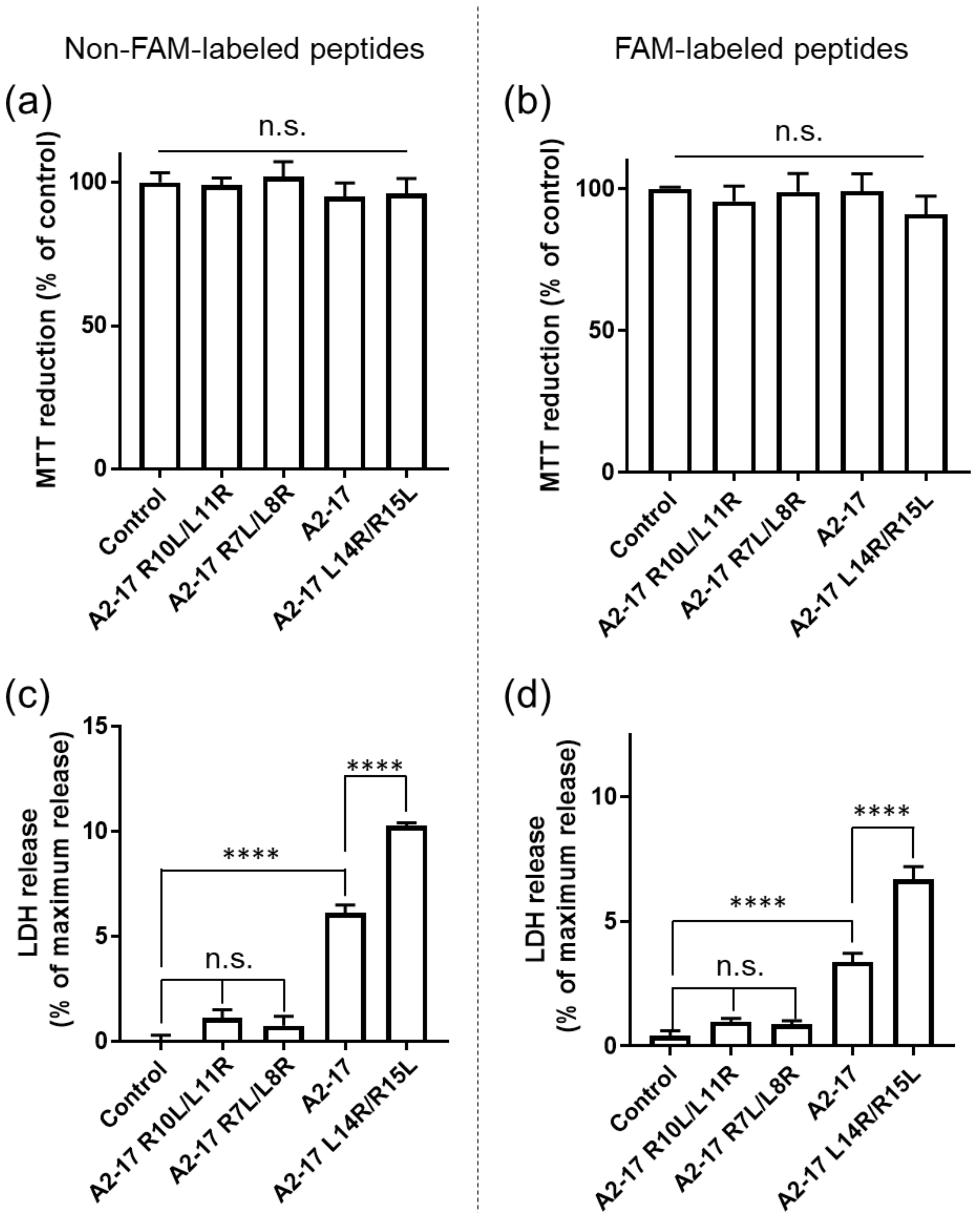

Figure S3. Cytotoxicity of A2-17 structural isomers to CHO-K1 cells after incubation with 2 μM of non-FAM-labeled peptides (a and c) or FAM-labeled peptides (b and d) for 30 min at 4℃. (a and b) The loss of the ability of cells to reduce MTT. (c and d) Membrane integrity determined by measuring the release of LDH in media. LDH released from cells lysed with 0.1 % Triton X-100 was defined as 100 % leakage. ****p < 0.0001; n.s., not significant.

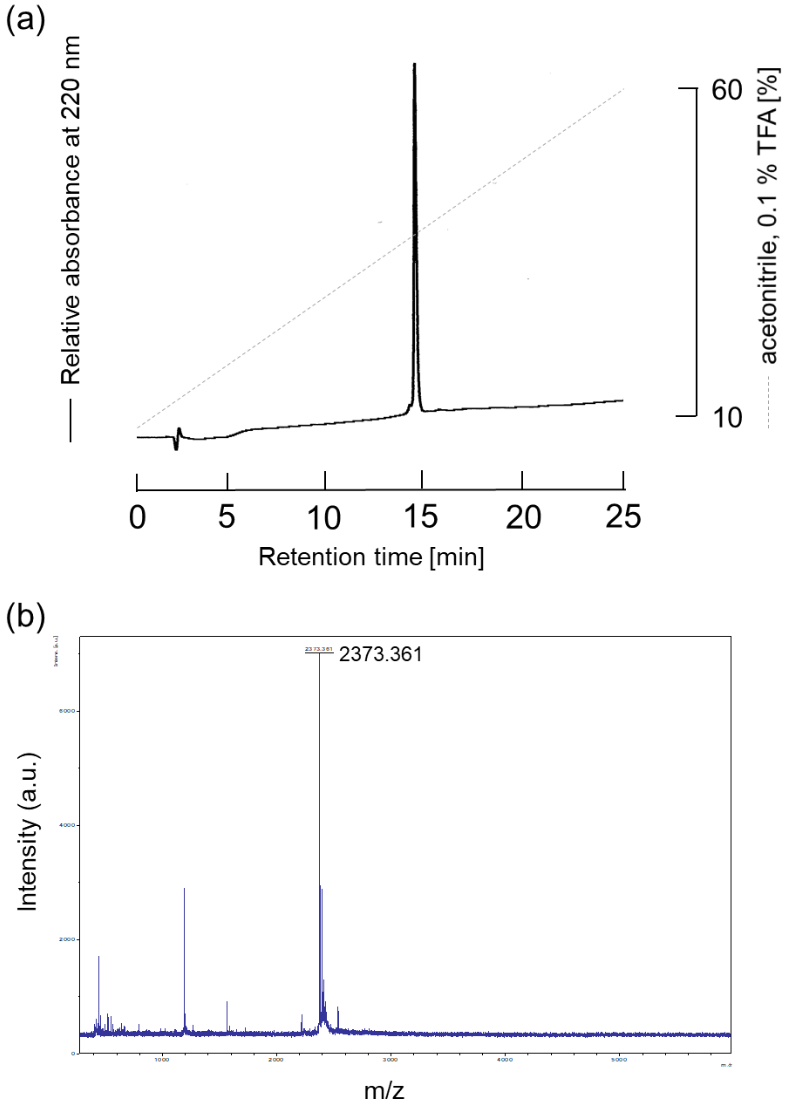

Figure S4. Characterization of A2-17 R10L/L11R. (a) Reverse-phase liquid chromatography profile. Column: COSMOSIL Packed Column 5C18-AR-ll (4.6x150 mm); eluent A: water, 0.1% trifluoroethanol (TFA); eluent B: acetonitrile, 0.1% TFA; gradient: 10-60% of eluent B/25 min; flow rate: 1.00 mL/min. The retention time was 14.4 min. (b) MALDI-TOF mass spectrometry. The identified exact mass was 2373.361 [calculated exact mass for $(M+H)^+$: 2373.6111].

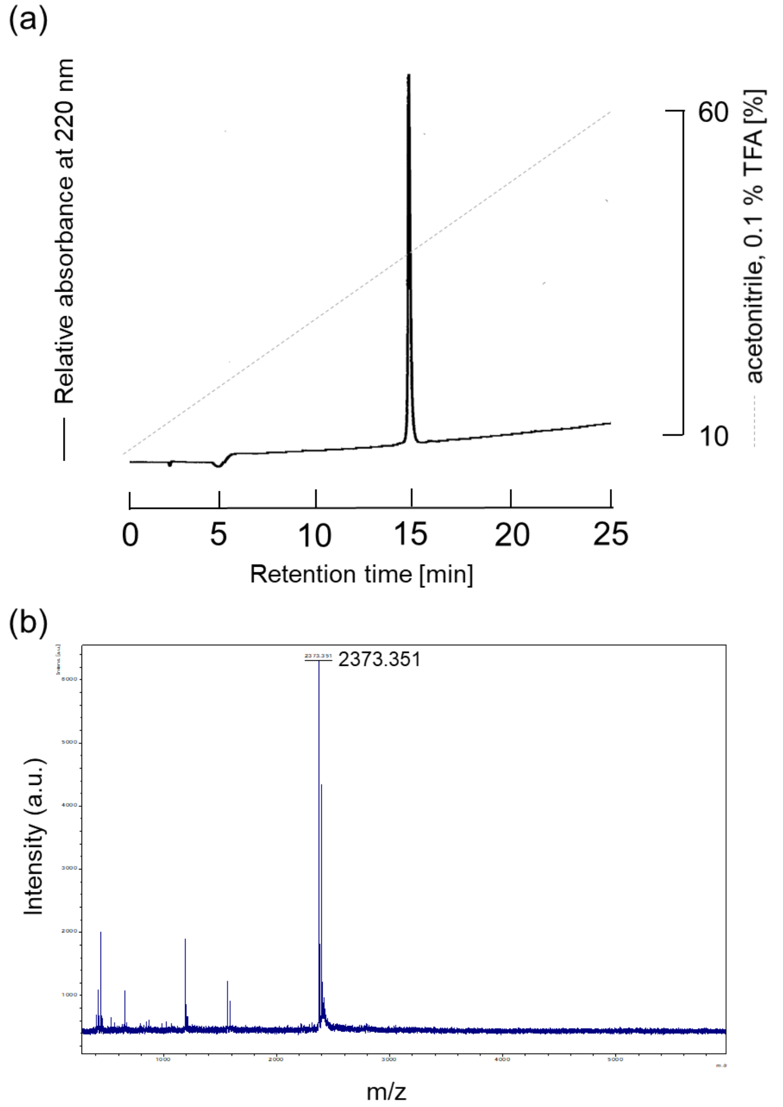

Figure S5. Characterization of A2-17 R7L/L8R. (a) Reverse-phase liquid chromatography profile. Column: COSMOSIL Packed Column 5C18-AR-ll (4.6x150 mm); eluent A: water, 0.1% TFA; eluent B: acetonitrile, 0.1% TFA; gradient: 10-60% of eluent B/25 min; flow rate: 1.00 mL/min. The retention time was 14.6 min. (b) MALDI-TOF mass spectrometry. The identified exact mass was 2373.351 [calculated exact mass for $(M+H)^+$: 2373.6111].

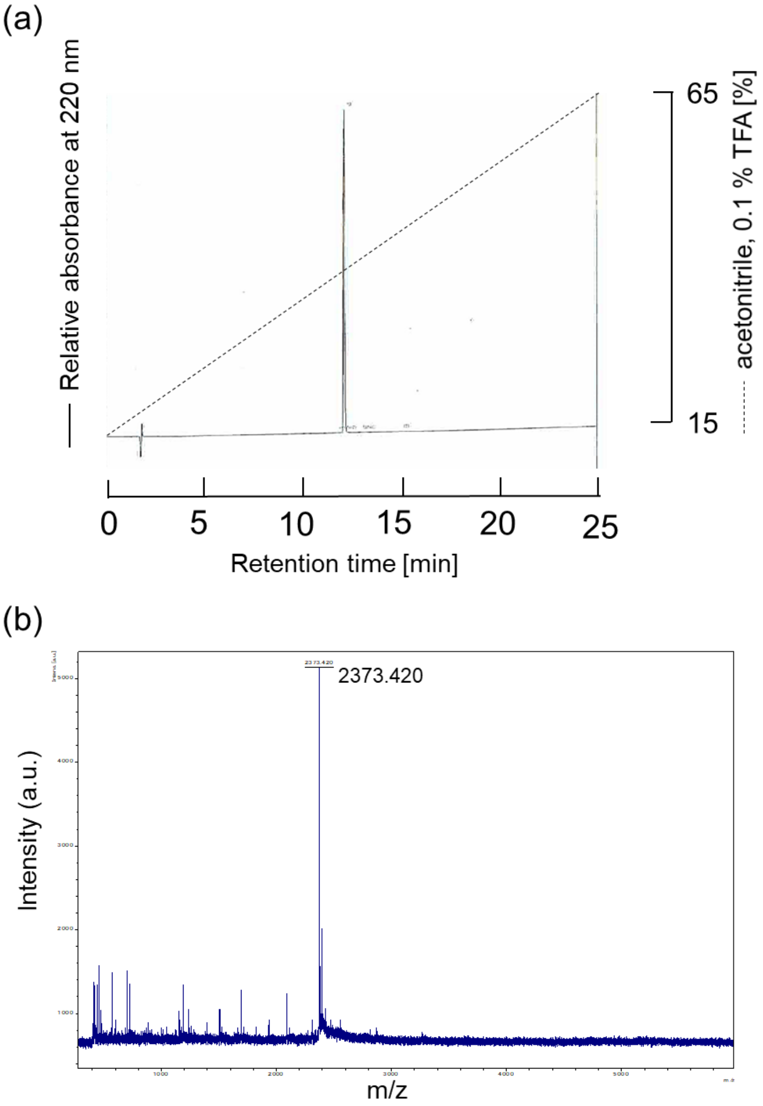

Figure S6. Characterization of A2-17. (a) Reverse-phase liquid chromatography profile. Column: Zorbax 300SB-C18 (4.6 x150 mm); eluent A: water, 0.1% TFA; eluent B: acetonitrile, 0.1% TFA; gradient: 15-65% of eluent B/25 min; flow rate: 1.00 mL/min. The retention time was 12.1 min. (b) MALDI-TOF mass spectrometry. The identified exact mass was 2373.420 [calculated exact mass for $(M+H)^+$: 2373.6111].

.

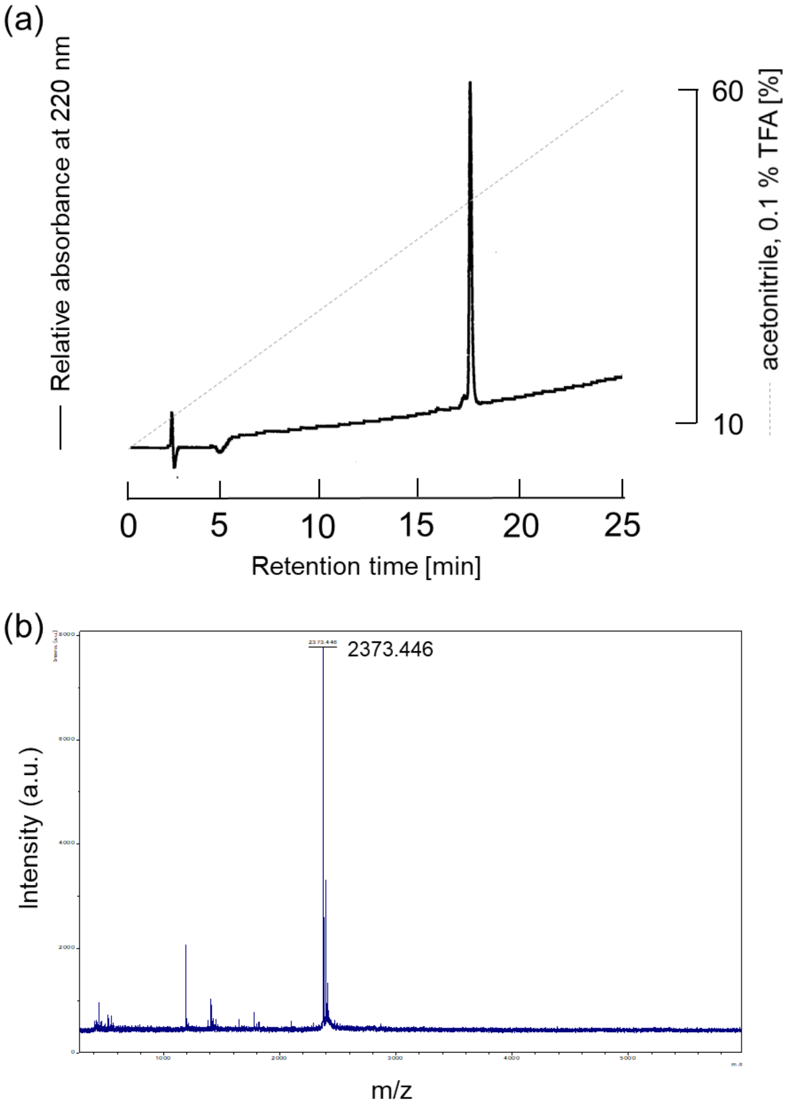

Figure S7. Characterization of A2-17 L14R/R15L. (a) Reverse-phase liquid chromatography profile. Column: COSMOSIL Packed Column 5C18-AR-ll (4.6x150 mm); eluent A: water, 0.1% TFA; eluent B: acetonitrile, 0.1% TFA; gradient: 10-60% of eluent B/25 min; flow rate: 1.00 mL/min. The retention time was 17.4 min. (b) MALDI-TOF mass spectrometry. The identified exact mass was 2373.446 [calculated exact mass for $(M+H)^+$: 2373.6111].

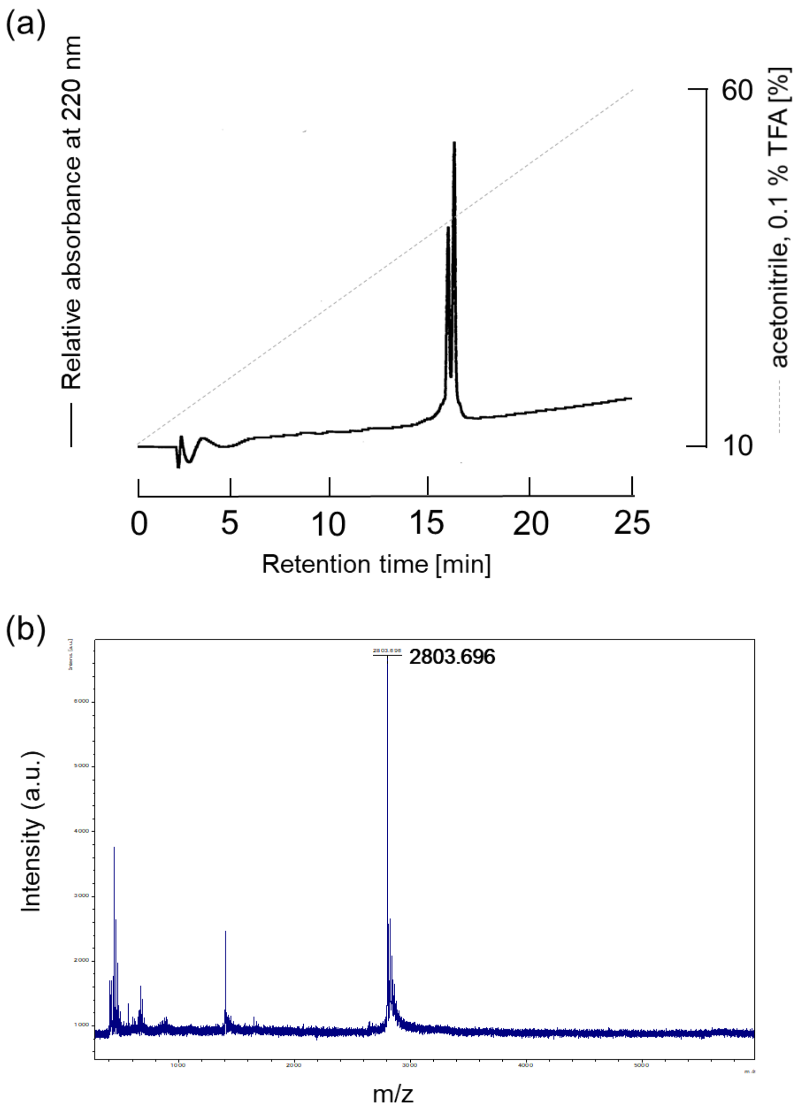

Figure S8. Characterization of FAM-labeled A2-17 R10L/L11R. (a) Reverse-phase liquid chromatography profile. FAM labeling of peptide gave a single doublet peak because the FAM used in this study is a mixture of 5-carboxyfluorescein and 6-carboxyfluorescein [*Bioconjugate Chem.* **14,** 653−660 (2003)]. Column: COSMOSIL Packed Column 5C18-AR-ll (4.6x150 mm); eluent A: water, 0.1% TFA; eluent B: acetonitrile, 0.1% TFA; gradient: 10-60% of eluent B/25 min; flow rate: 1.00 mL/min. The retention time was 14.7-15.7 min. (b) MALDI-TOF mass spectrometry. The identified exact mass was 2803.696 [calculated exact mass for $(M+H)^+$: 2803.6908].

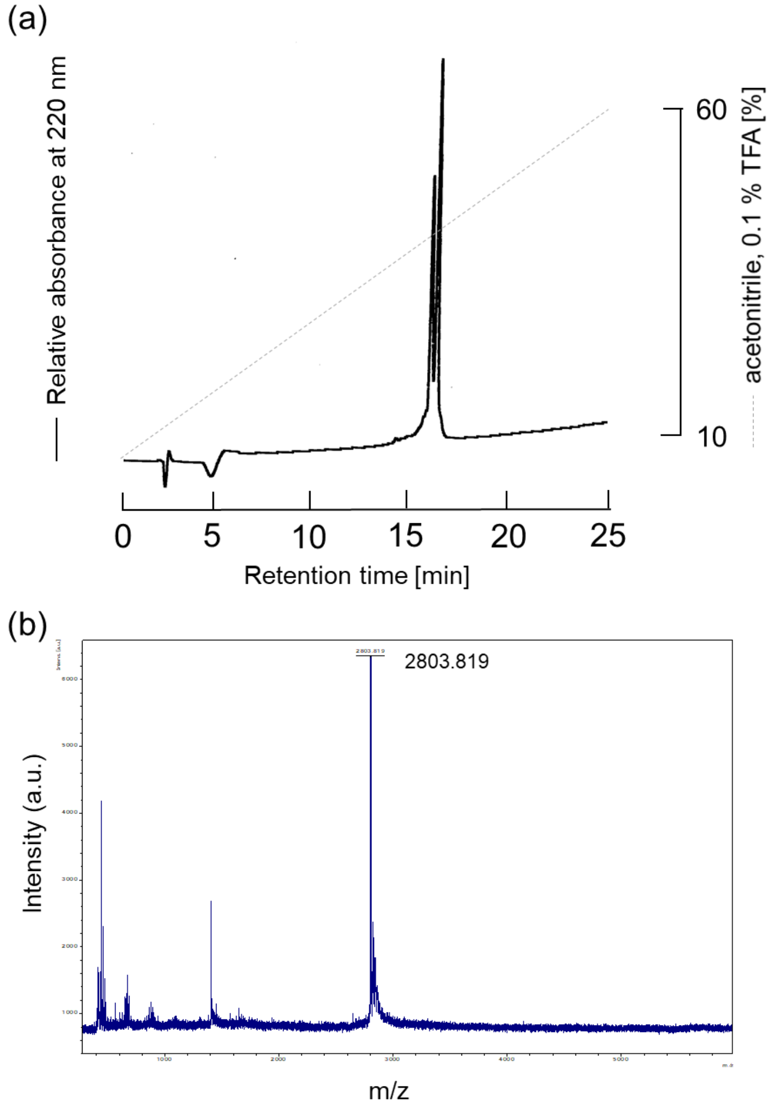

Figure S9. Characterization of FAM-labeled A2-17 R7L/L8R. (a) Reverse-phase liquid chromatography profile. FAM labeling of peptide gave a single doublet peak because the FAM used in this study is a mixture of 5-carboxyfluorescein and 6-carboxyfluorescein [*Bioconjugate Chem.* **14,** 653−660 (2003)]. Column: COSMOSIL Packed Column 5C18-AR-ll (4.6x150 mm); eluent A: water, 0.1% TFA; eluent B: acetonitrile, 0.1% TFA; gradient: 10-60% of eluent B/25 min; flow rate: 1.00 mL/min. The retention time was 15.7-17.4 min. (b) MALDI-TOF mass spectrometry. The identified exact mass was 2803.819 [calculated exact mass for $(M+H)^+$: 2803.6908].

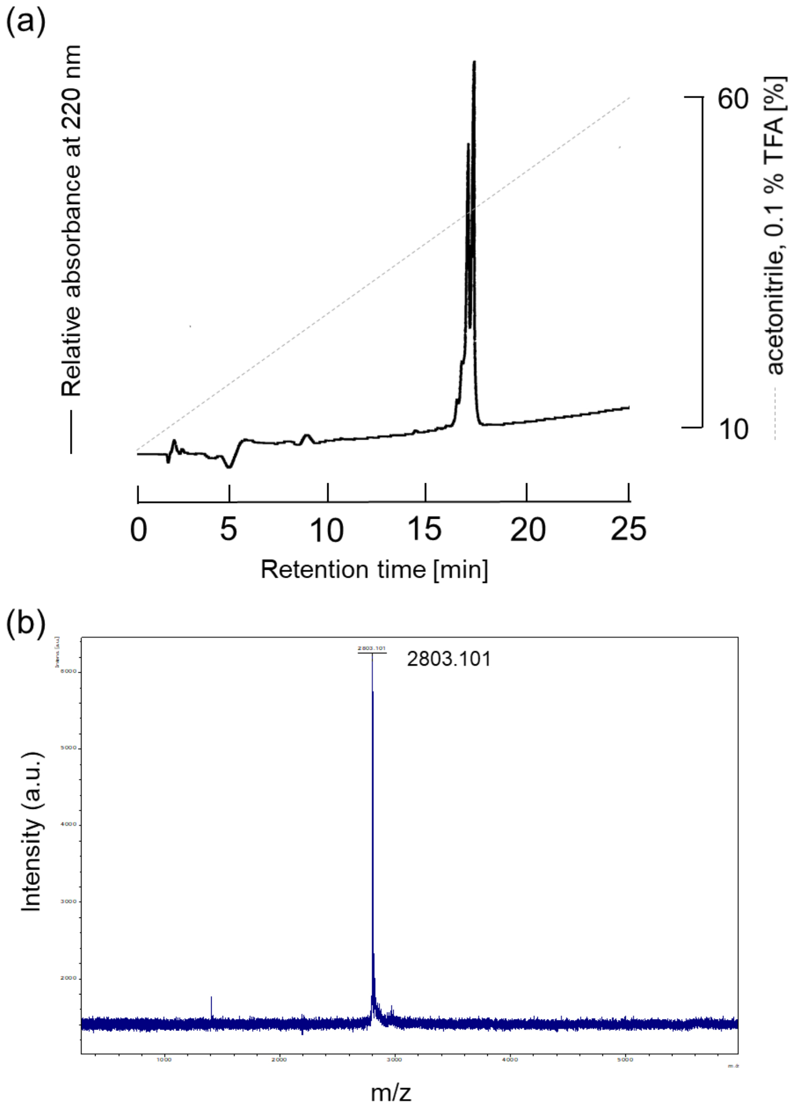

Figure S10. Characterization of FAM-labeled A2-17. (a) Reverse-phase liquid chromatography profile. FAM labeling of peptide gave a single doublet peak because the FAM used in this study is a mixture of 5-carboxyfluorescein and 6-carboxyfluorescein [*Bioconjugate Chem.* **14,** 653−660 (2003)]. Column: COSMOSIL Packed Column 5C18-AR-ll (4.6x150 mm); eluent A: water, 0.1% TFA; eluent B: acetonitrile, 0.1% TFA; gradient: 10-60% of eluent B/25 min; flow rate: 1.00 mL/min. The retention time was 16.2-16.7 min. (b) MALDI-TOF mass spectrometry. The identified exact mass was 2803.101 [calculated exact mass for $(M+H)^+$: 2803.6908].

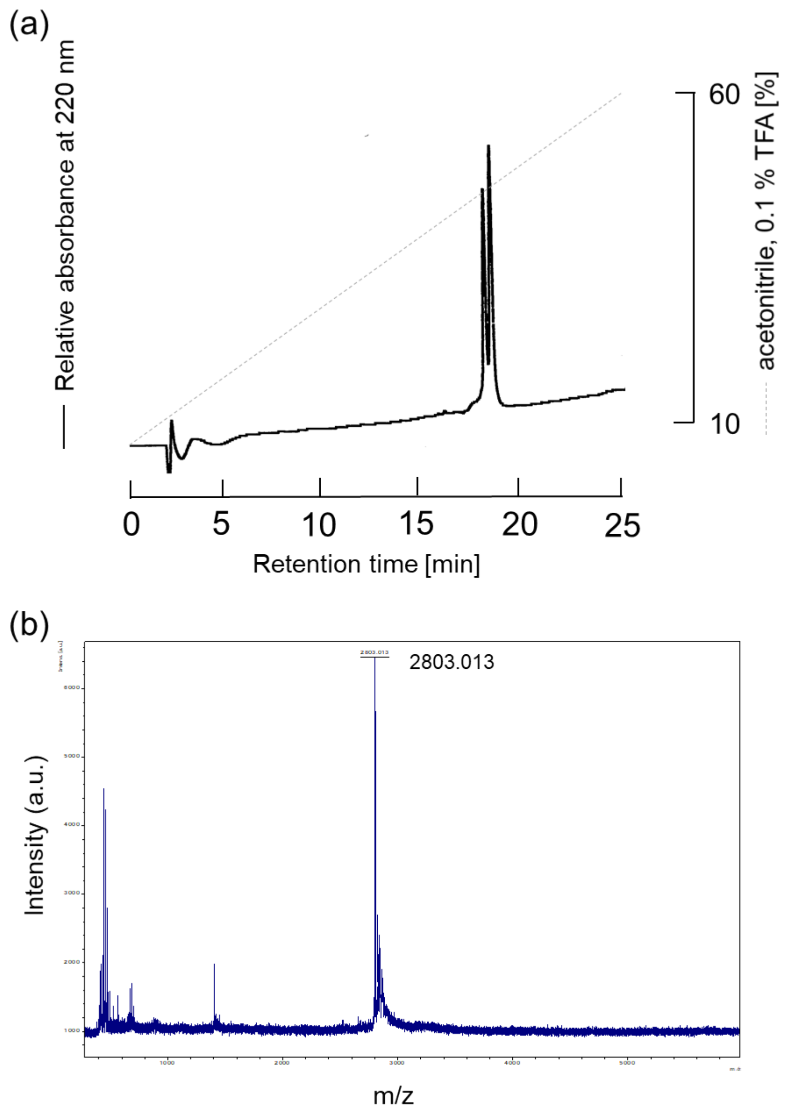

Figure S11. Characterization of FAM-labeled A2-17 L14R/R15L. (a) Reverse-phase liquid chromatography profile. FAM labeling of peptide gave a single doublet peak because the FAM used in this study is a mixture of 5-carboxyfluorescein and 6-carboxyfluorescein [*Bioconjugate Chem.* **14,** 653−660 (2003)]. Column: COSMOSIL Packed Column 5C18-AR-ll (4.6x150 mm); eluent A: water, 0.1% TFA; eluent B: acetonitrile, 0.1% TFA; gradient: 10-60% of eluent B/25 min; flow rate: 1.00 mL/min. The retention time was 16.9-18.1 min. (b) MALDI-TOF mass spectrometry. The identified exact mass was 2803.013 [calculated exact mass for $(M+H)^+$: 2803.6908].
